# Amount of reoperation following surgical repair of nonsyndromic craniosynostosis at a single center

**DOI:** 10.55730/1300-0144.5428

**Published:** 2022-05-07

**Authors:** Duygu BAYKAL, Rabia Nur BALÇIN, Mevlüt Özgür TAŞKAPILIOĞLU

**Affiliations:** 1 Department of Neurosurgery, Bursa State Hospital, Bursa, Turkey; 2 Department of Neurosurgery, Faculty of Medicine, Bursa Uludağ University, Bursa, Turkey

**Keywords:** Craniosynostosis, reoperation, nonsyndromic craniosynostosis

## Abstract

**Background/aim:**

Craniosynostosis is a deformity of the skull that occurs as a result of early fusion of one or more cranial sutures and can be accompanied by neurological deficits. Craniosynostosis can be classified as syndromic or nonsyndromic according to the type of suture involved. Surgical treatment of craniosynostosis in infants basically involves loosening and opening the fused sutures to reduce intracranial pressure, allow the brain to grow, and also fix the skull shape. However, in such cases there is a risk of resynostosis after surgery. According to the literature, resynostosis rates vary between 0% and 70%. In this study, we aimed to evaluate the reoperation rate in craniosynostosis cases treated surgically in our clinic.

**Material and methods:**

A retrospective analysis of 70 nonsyndromic craniosynostosis cases treated surgically in the Neurosurgery Department of Bursa Uludağ University from 2005 to 2019 was performed. All patients had undergone total cranial vault remodeling surgically and had been followed up for at least a year.

**Results:**

The study group included 70 patients, comprising 40 (57.1%) male and 30 (42.9%) female patients. The mean age of the group was 10.9 ± 7.8 months (range 3–34 months). Out of 70 patients, repeat surgery due to resynostosis had been performed once in 5 (7.1%) patients and twice in 1 (1.4%) patient.

**Conclusion:**

It should be kept in mind that resynostosis may occur in patients who have been operated for craniosynostosis. Patients should be examined cosmetically and if necessary, radiologically in the follow-up. Further studies based on larger sample size are recommended for more quantitative data and better results.

## 1. Introduction

The term craniosynostosis was coined by Otto in 1830, who defined it as the premature fusion of cranial sutures leading to cranial deformity. In 1851, Virchow laid down the general rules for explaining the cranial deformities accompanying craniosynostosis that resulted in interrupted growth and compensatory changes of the skull. Although craniosynostosis is considered to be primarily a developmental defect originating in utero or immediately after birth, several theories have been put forth to explain the etiology of this condition. Advancements in this field have resulted in the identification of genetic mutations related to craniosynostosis syndromes, which has in turn facilitated a better understanding of the mechanisms and factors related to this condition [1].

There exist several classification systems for craniosynostosis. First, depending on the number of sutures undergoing premature fusion, craniosynostosis is termed as simple (a single suture) or complex (two or more sutures). Second, the condition is categorized as primary if it is a case of isolated craniosynostosis or secondary if it occurs along with comorbidities such as hematological or metabolic disorders, hyperthyroidism, etc. Lastly, the condition is classified as syndromic if it involves multiple organs or nonsyndromic if it is an isolated case of craniosynostosis, which is the most common anomaly [2].

Nonsyndromic craniosynostosis is observed in 80%–85% of the cases. Among the different types of nonsyndromic craniosynostosis, the most common is scaphocephaly (fusion of the sagittal suture), which constitutes 40%–60% of all cases. The others include trigonocephaly (fusion of the metopic suture), posterior plagiocephaly (fusion of the lambdoid suture), brachycephaly (fusion of the bicoronal suture), and anterior plagiocephaly (fusion of the unicoronal suture) [3]. Syndromic craniosynostosis, like Apert, Crouzon, Muenke, Pfeiffer, Saethre-Chotzen, affects up to 1:30,000 live births with characteristic craniofacial growth restrictions, deformities, and other associated abnormalities. More than 150 syndromes are associated with craniosynostosis.

The fundamental goal of surgical management of craniosynostosis is to loosen the fused sutures for the creation of sufficient space in the cranial vault for the brain to grow, prevent the complications of excessive intracranial pressure, and fix the skull shape. Although there are several surgical approaches such as endoscopic suturectomy, spring-assisted surgery, and total cranial vault remodeling. Total cranial vault remodeling with an optimum surgical duration of 2–12 months, is most commonly used in various centers [4].

The rates of reoperation are not clearly defined in the literature. They have been reported to vary between 0% and 70%. There are contradictory reports with regard to the most frequently noted resynostosis based on the type of sutures involved. Some authors state that it is most common in sagittal sutures [5] whereas others maintain that it is least common in these sutures [6]. Hence, we aimed to analyze the reoperation rates in the craniosynostosis cases that were surgically treated in our clinic.

## 2. Material and methods

All procedures performed in studies involving human participants were in accordance with the ethical standards of the institutional and/or national research committee (Institutional Review Board of Bursa Uludağ University 2020–21/1) and with the 1964 Helsinki Declaration and its later amendments or comparable ethical standards.

A retrospective analysis of 70 infants who had been operated upon for nonsyndromic craniosynostosis between 2005 and 2019 in the Neurosurgery Department of the Medical Faculty of Bursa Uludağ University was conducted. All of them had undergone total cranial vault remodeling surgery and had been followed up for at least a year.

### 2.1 Statistical analysis

The conformity of the data to the normal distribution was tested with the Shapiro-Wilks tests. Descriptive statistics are given as median (minimum:maximum) values, and descriptive statistics of categorical data are given as frequency and percentage. Fisher-Freeman-Halton test was performed for comparing sex distribution among study groups and the Kruskal-Wallis test was also performed for comparing age(M), time of operation(min), amount of bleeding(cc), amount of transfusion (cc), time of hospitalization(d). SPSS version 23.0 (SPSS Inc., Chicago, IL, USA). Dunn-Bonferroni test was also performed for pairwise comparisons after the Kruskal-Wallis test. A p-value of < 0.05 was considered statistically significant for all comparisons.

### 2.2. Surgical procedure

Depending on the closed suture, the patient was operated in the prone or supine position. A bicoronal incision was made through the anterior and posterior fontanelle and the skin flaps were elevated up to the supraorbital rim anteriorly and external occipital tubercle posteriorly. The periosteum was dissected and a biparietal craniotomy was performed. During this process, extreme caution was exercised to avoid any bleeding. The temporal muscles were peeled up to the squamous part of the bilateral temporal bones. Four bilateral burr-holes were made 2–3 cm lateral to the midline. Depending on the severity of the scaphocephaly, sagittal suture excision was performed, and the skull was reshaped with “π” osteotomies. The sagittal suture was cut at its edges in the form of a bar and separated from the dural sinuses. Barrel stave osteotomies were performed on the frontal and occipital bones at equal intervals to increase the intertemporal width and broaden the occipital region. A “π” shape was created after removing 2 additional bone bars from the bilateral temporal bones. A curvilinear bone incision extending inferiorly and superiorly from the anterior leg of the “π” was made to increase the biparietal diameter and loosen the temporal bone. The resulting bone flaps were stretched outwardly. A silicon drain was placed in the surgical area to provide drainage to the entire epidural space. The bone flaps were fixed to the sagittal suture with several 2–0 nonabsorbable sutures, and the wound was closed in layers.

Patients with coronal or metopic synostosis were treated with anterior cranial vault remodeling and fronto-orbital advancement. The patients with lambdoid synostosis were treated with posterior cranial vault remodeling, which included excision of the fused suture. Patients with multiple-suture involvement were operated on with the combination of the aforementioned procedures.

## 3. Results

The 70 craniosynostosis cases comprised 32 (45.7%) scaphocephaly, 24 (34.3%) trigonocephaly, 6 (8.6%) plagiocephaly, 5 (7.1%) brachycephaly, and 3 (4.3%) pansynostosis cases.

There was a male predominance among the patients who underwent surgery, as there were 40 (57.1%) males and 30 (42.9%) females. The mean age of the entire study group was 10.9 ± 7.8 months (range 3–34 months). The mean values of the various parameters related to the surgery are as follows: surgical duration was 108 ± 37 min (range 60–225 min), estimated blood loss was 66.7 ± 37.2 mL (range 10–150 mL), intraoperative blood transfusion requirement was 72.5 ± 48.3 mL (range 0–220 mL), and length of stay in the hospital was 2.6 ± 2.2 days (range 1–12 days) (Table 1).

While age, sex and time of operation did not differ between the groups (p > 0.05), it was determined that there was a difference in the study groups according to the amount of bleeding and amount of transfusion (p = 0.033 and p = 0.031 retrospectively). However, in the subgroup analyzes, no significant results could be obtained because the number of units in the groups was not sufficient to reveal the significance in pairwise comparisons.

Out of the 70 patients, 5 (7.1%) of them (4 had scaphocephaly and 1 had brachycephaly) underwent repeat surgery only once and 1 (1.4%) patient (had scaphocephaly) underwent the procedure twice due to resynostosis (Figures a,b,c) (Table 2). The time interval between the surgeries of the patients was 13.3 ± 6.6 months – the patient who underwent repeat surgery twice due to resynostosis had the first one 6 months after the initial surgery and the second one 12 months later. Among the infants who underwent repeat surgery, the mean surgical duration, estimated blood loss, intraoperative blood transfusion requirement, and length of stay in the hospital were 100.8 ± 9.7 min (range 90–115 min), 50.0 ± 36.3 cc (10–110 cc), 55.0 ± 46.3 cc (0–130 cc), 3.8 ± 0.7 days (range 3–5 days), respectively. No intraoperative or postoperative complications or mortality were recorded in any of the cases.

## 4. Discussion

Craniosynostosis not only results in cosmetic deformities but also impacts the growth and development of the child, including speech, behavior, and psychology. The reoperation rates mentioned in the literature vary. Wall et al. reported the reoperation rates for single suture synostosis cases to be 5.2% (5 out of 97) [7]. McCarthy et al. reported this rate in nonsyndromic patients as 13.5% (14 out of 104) [8]. In our study, among the 70 patients who had been operated upon, 6 (8.5%) underwent repeat surgery due to resynostosis. Therefore, the operation rates due to resynostosis in nonsyndromic patients in this study are comparable with the rates mentioned in the literature.

The exact mechanism of early reclosure of the sutures is unknown. In an experimental model Hermann et al. concluded that bone regeneration in the cranium is both age and location dependent [9]. Postoperative calvarial growth restriction because of fibrosis of newly formed bone and pericranium may cause resynostosis [10]. The other theory is resynostosis due to underlying disorder that caused the first synostosis [11]. Bone morphogenetic proteins are known to be expressed during normal bone healing. Over expression of these proteins may be another possible mechanism of resynostosis [12].

Several studies till date have focused on specific types of craniosynostosis, and the rates of reoperation mentioned in the literature greatly vary. Wagner et al., who studied nonsyndromic bicoronal synostosis cases, reported that 36% of them needed reoperation [13]. Another study focusing on isolated sagittal synostosis reported the reoperation rate to be as high as 16.7% [14]. In our case, it was 20% and 12.5% for bicoronal and sagittal synostosis, respectively. This huge difference in the resynostosis rates between other studies and ours could be attributed to the fact that we included only patients who underwent repeat surgery in our study and excluded resynostosis cases that did not require surgery.

The resynostosis rates have been reported to be higher in syndromic patients compared to nonsyndromic patients, in the literature [5]. Foster et al. reported in their study that multiple-suture synostosis was noted in 50% of the cases with total resynostosis of which, 37.5% had sagittal suture synostosis. Proven syndromic cases were excluded from our study; however, multiple-suture synostosis was noted in 2 cases. No evidence of syndromic craniosynostosis was found on genetic analysis and physical examination of these patients and also no resynostosis was detected.

An increase in the intracranial pressure before the first operation in about 50% of the patients with resynostosis has been reported in earlier studies [15,16]. It was suggested that intracranial pressure measurement in craniosynostosis may be helpful, which cannot be determined clearly because of wide variations of the disease [5]. In our study, no intracranial pressure measurement of resynostosis cases was performed. Diagnosis and treatment were based on the computed tomography scan results and clinical condition of the patients.

Wall et al. reported higher reoperation rates in infants who underwent primary surgery before 6 months of age than those who underwent surgery later [7]. However, several studies in the literature could not find any correlation between reoperation rate and the age at which the patient underwent surgery [5,17]. In our study, the mean age of the patients who underwent their first surgery due to craniosynostosis was 10.9 ± 7.8 months. No correlation between the age at first operation and reoperation was found in this study. However, while determining the appropriate time for the first surgery, it must be kept in mind that craniosynostosis is a progressive deformity of the skull base which may be difficult to fix later.

As various studies in the literature have used numerous surgical techniques and reported a wide variation in the reoperation rate, it is difficult to form a correlation between the type of primary surgery and reoperation [5]. Foster et al. reported no difference in the mean increase in head circumference between the groups with and without resynostosis. Additionally, they demonstrated that enlargement of the skull following the first operation was not related to reoperation [5]. In our study, with the exception of 2 cases with pansynostosis, the head circumference correlated with the age. During the follow-up after surgery, resynostosis was detected due to cosmetic deformity.

When we compare the reoperated and single operated patients clinically and radiologically there were no difference at early follow-up period; but significant head deformity was observed in further follow-ups pointing the premature reclosure of the sutures and there was clear evidence of early closure at CT scans. There was no neurological difference between two patient groups.

One of the main limitations of this study was the small population size. Cases with early onset were operated earlier while late-onset and mild cases underwent surgery at an older age. All the patients were operated by the same surgeon. Therefore, if there was a technical insufficiency, it might have affected the results.

Although neurological disorders were not detected in the reoperated patients in our series, early detection and intervention followed by developmental monitoring are vital for improving the chances of infants with craniosynostosis and reducing its associated risks, including developmental delay. A multispecialty-team approach involving orthopedics, pediatrics, neurosurgery, and plastic surgery should be considered for better management of such patients.

## Figures and Tables

**Figure:**
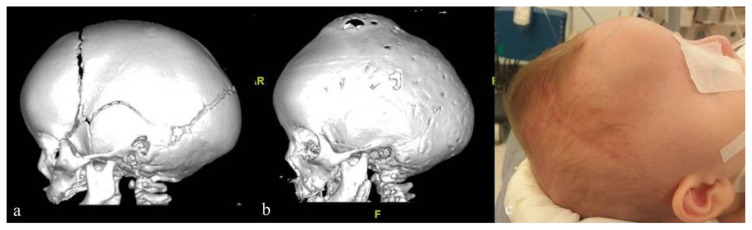
Case 6: 6 months old scaphocephaly patient. a: Image of first preoperative 3D CT, b: Image of second preoperative 3D CT, c: Photography before the second surgery shows bony bulging of vertex.

**Table 1:** All operated nonsyndromic craniosynostosis cases.

Type of Craniosynostosis	No. (%)	Sex	Age (M)	Time of operation (min)	Amount of bleeding (cc)	Amount of transfusion (cc)	Time of hospitalization(d)
Scaphocephaly	32(%45.7)	19 M, 13F	10(3:75)	105(60:225)	50(10:200)	55(0:220)	2(0:19)
Trigonocephaly	24(%34.3)	15 M, 9 F	9(4:22)	120(75:180)	90(30:300)	90(30:400)	2(1:5)
Plagiocephaly	6(%8.6)	3 M, 3 F	8.50(6:34)	120(75:180)	60(10:80)	50(0:90)	2(1:6)
Brachycephaly	5(%7.1)	2 M, 3 F	8(3:17)	120(90:180)	50(20:120)	50(20:120)	2(1:3)
Pansynostosis	3(%4.3)	1 M, 2 F	12(6:37)	120(60:270)	60(30:190)	60(30:200)	3(3:4)
p-value	70(%100)	0,807^a^	0.780^b^	0.076^b^	**0.033** b	**0.031** b	0.882^b^
		**Pairwise comparisons**					
		**P** **_12_**	**P** **_13_**	**P** **_14_**	**P** **_23_**	**P** **_24_**	**P** **_34_**
Amount of bleeding(cc)		0.083	>0.99	>0.99	0.253	0.425	>0.99
Amount of transfusion (cc)		0.112	>0.99	>0.99	0.186	0.345	>0.99

M: moths, min: minutes, cc: cubic cm, d: day

Data were presented as median (minimum:maximum) and n%.

The pansynostosis group was not included in the analysis due to insufficient sample size (n = 3).

a Fisher-Freeman-Halton Test,

b Kruskal-Wallis Test

P_12_: P_Scaphocephaly vs. Trigonocephaly_, P_13_: P_Scaphocephaly vs. Plagiocephaly_, P_14_: P_Scaphocephaly vs. Brachycephaly_, P_23_: P _Trigonocephaly vs. Plagiocephaly_, P_24_: P _Trigonocephaly vs. Brachycephal_, P_34_: P _Plagiocephaly vs. Brachycephal_,

**Table 2:** All operated resynostosis cases.

No	Type of craniosynostosis	Time of recurrence (month)	Time of operation (min)	Amount of bleeding (mL)	Amount of transfusion (mL)	Time of hospitalization (day)
1	Brachycephaly	12	115	110	130	5
2	Scaphocephaly	18	100	10	0	4
3	Scaphocephaly	9	90	20	20	3
4	Scaphocephaly	24	105	70	60	3
5	Scaphocephaly	12	90	50	80	4
6	Scaphocephaly	7	105	40	40	4
7(2)	Scaphocephaly	12	90	90	100	4

7(2): second operation of #6
